# IMPACT OF AN ONLINE SOCIAL INTELLIGENCE TRAINING PROGRAM ON CUSTODIAL GRANDMOTHERS’ WELL-BEING

**DOI:** 10.1093/geroni/igac059.1166

**Published:** 2022-12-20

**Authors:** Megan Dolbin-MacNab, Gregory Smith, Frank Infurna, Britney Webster, Luxin Hu, Saul Castro, Max Crowley, Carol Musil

**Affiliations:** Virginia Tech, Blacksburg, Virginia, United States; Kent State University, Stow, Ohio, United States; Arizona State University, Phoenix, Arizona, United States; Kent State University, Kent, Ohio, United States; Kent State University, Kent, Ohio, United States; Arizona State University, Phoenix, Arizona, United States; Pennsylvania State University, University Park, Pennsylvania, United States; Case Western Reserve University, Cleveland, Ohio, United States

## Abstract

Despite widespread evidence that the circumstances leading to care, combined with the stress of full-time parenting, have profound effects on psychological, physical, and social functioning of custodial grandmothers (CGM) and their adolescent grandchildren (GC), evidence-based interventions for these families are scarce. To address this gap, we conducted a randomized clinical trial (RCT) with 349 nationally recruited CGMs which compared an online social intelligence training intervention (SIT; n=185) to an attention-control (AC; n=164) condition. The SIT focused on enhancing CGMs’ capacity to develop and sustain positive social ties; an important goal since working models of attachment and caregiving are challenged and re-shaped by the off-time and demanding nature of parenting a GC. To investigate initial impact of SIT, we conducted multi-domain latent difference score models (Mplus 8) on a full intent-to-treat basis comparing the two RCT conditions on changes across key outcomes from baseline to immediate post-intervention. In comparison to AC, SIT yielded statistically significant improvement in CGMs’ depressed affect, self-esteem, relationship quality with the GC, and attachment avoidance and attachment anxiety with GC. Contrary to expectations, no significant differences were found between the two conditions on outcomes indicative of social competence (e.g., perspective taking, social awareness, social information processing, social self-monitoring). We conclude that CGMs may have applied information obtained from the SIT primarily to their relationship with GC. Our findings point to the potential benefits of the self-guided SIT, given that it can be delivered online and therefore has widespread reach to a vulnerable population. [Funded by R01AG054571]

